# Effects of *Lippia citriodora* Leaf Extract on Lipid and Oxidative Blood Profile of Volunteers with Hypercholesterolemia: A Preliminary Study

**DOI:** 10.3390/antiox10040521

**Published:** 2021-03-27

**Authors:** Antonella Angiolillo, Deborah Leccese, Marisa Palazzo, Francesco Vizzarri, Donato Casamassima, Carlo Corino, Alfonso Di Costanzo

**Affiliations:** 1 Centre for Research and Training in Medicine of Aging, Department of Medicine and Health Sciences “V. Tiberio”, University of Molise, 86100 Campobasso, Italy; d.leccese@studenti.unimol.it (D.L.); alfonso.dicostanzo@unimol.it (A.D.C.); 2 Department of Agricultural, Environmental and Food Sciences, University of Molise, 86100 Campobasso, Italy; casamassima.d@unimol.it; 3 Department of Agricultural and Environmental Science, University of Bari Aldo Moro, 70126 Bari, Italy; francesco.vizzarri@uniba.it; 4 Department of Veterinary Medicine, University of Milano, 26900 Lodi, Italy; carlo.corino@unimi.it

**Keywords:** verbascoside, hypercholesterolemia, antioxidants

## Abstract

*Lippia citriodora* is a plant traditionally used for its anti-inflammatory, antioxidant and antispasmodic effects, as well as for additional biological activities proven in cell culture, animal studies and a small number of human clinical trials. The plant has also shown a marked improvement in blood lipid profile in some animal species. In the present preliminary study, we investigated the effect of a leaf extract on lipid and oxidative blood profile of hypercholesterolemic volunteers. Twelve adults received *Lippia citriodora* extract caps, containing 23% phenylpropanoids, (100 mg, once a day) for 16 weeks. Selected blood lipids and plasma oxidative markers were measured at baseline and after 4, 8 and 16 weeks of treatment. Compared with baseline, total cholesterol levels significantly decreased and high-density lipoprotein cholesterol increased, while low-density lipoprotein cholesterol and triglycerides showed only a downward trend. Oxidative status was improved due to a decrease in the concentration of total oxidant status, reactive oxygen metabolites and malondialdehyde, and a significant increase in ferric reducing ability of plasma, vitamin A and vitamin E. These preliminary results suggest that dietary supplementation with *Lippia citriodora* extract can improve the lipid profile, enhance blood antioxidant power, and could be a valuable natural compound for the management of human hypercholesterolemia.

## 1. Introduction

Cardiovascular disease (CVD) is a pathological process that affects the arterial system as a whole and determines the progressive narrowing of the arteries, up to their complete obstruction. Therefore, it must be considered a unique disease that manifests itself clinically in different ways, depending on which arterial district is concerned. In Western countries, it still represents the main cause of death and an important contribution to disability. The prevalence of CVD cases nearly doubled from 1990 to 2019, and the number of CVD deaths in the same period has steadily increased [1].

The principal risk factors converge on an unhealthy diet, typical of industrialized countries, with consequent dyslipidemia, diabetes, obesity and hypertension. Additional factors include a sedentary lifestyle, stress and smoking, which, unfortunately, are still widespread. High levels of low-density lipoprotein cholesterol (LDL-C) are one of the main modifiable cardiovascular risk factors (CVRF) [1]. Indeed, LDL-C and other cholesterol-rich apolipoprotein-B-containing lipoproteins, including very low-density lipoproteins (VLDL) and their remnants, intermediate-density lipoproteins (IDL) and lipoprotein (a), play a pivotal role in the development of atherosclerotic plaques since, in high concentrations, they accumulate within the arterial intima [2]. At this site they are separated from plasma antioxidants and become particularly susceptible to oxidative alterations, acquiring proinflammatory and immunogenic properties. Although advanced lesions can grow enough to arrest blood flow, the main clinical complication is an acute obstruction due to a thrombus development [3,4]. LDL-C concentration is closely linked to the incidence of atherosclerotic CVD, such as myocardial infarction and ischemic stroke [2].

Evaluation of the cumulative effect of the various CVRF and estimation of atherosclerotic CVD risk is crucial to the implementation of prevention programs. Recent guidelines of the European Society of Cardiology (ESC) and the European Atherosclerosis Society (EAS) for the management of dyslipidemias provide Systematic Coronary Risk Evaluation (SCORE) charts, which indicate the risk of developing atherosclerotic CVD over the next ten years. The risk is calculated considering age, sex, smoking status, systolic blood pressure and total cholesterol (TC). They also recommend new LDL-C treatment goals based on cardiovascular risk categories (<116, 100, 70 and 55 mg/dL for low, moderate, high, and very high risk, respectively). The guidelines point out the importance of adopting and sustaining a healthy lifestyle, and preventative action for a person should be related to the total risk: the higher the risk, the more intense the action. In some cases, unfortunately, prevention is not sufficient and it is necessary to resort to the use of pharmacologic therapy [5]. The backbone drugs for hypercholesterolemia are statins. Their mechanism of action is focused on the competitive inhibition of the 3-hydroxy-3methyl-glutaryl coenzyme A (HMG-CoA) reductase, which regulates the limiting step of the synthesis of cholesterol at the level of hepatocytes; this leads to a reduction in cholesterol synthesis and an increase in LDL receptors at the level of liver cells, with a consequent further reduction in the plasma cholesterol. They produce several other effects termed as pleiotropic effects of statins [6]. If they are contraindicated, cholesterol-absorption inhibitors and bile-acid-binding resins can be used. In case of failure, an association therapy or monoclonal antibodies anti-proprotein convertase subtilisin/kexin type 9 or bempedoic acid can be assessed [5]. However, these drugs are not free of adverse effects: gastrointestinal disorders, myalgias, arthralgias, transaminases/creatine phosphokinase (CPK) elevation and rhabdomyolysis are common [7]. The guidelines also suggest a nutraceutical approach (that can or cannot include statins) which could help treat hypercholesterolemia whilst avoiding every possible side effect [8].

In the last few years attention was paid to several plants’ natural molecules, some of which can improve lipid profile and oxidative status [9,10]. *Lippia citriodora* (lemon verbena) is a plant from the Verbenaceae family, which grows spontaneously in South America and is cultivated in North Africa and South Europe. It is mainly used as a spice, but also as a medicinal plant possessing digestive, antispasmodic, antipyretic, anti-inflammatory, antioxidant, anxiolytic, neuroprotective, anticancer, anesthetic, antimicrobial and sedative properties. Leaf infusions have traditionally been used to treat cold, fever, colic, diarrhea, nerve problems, acne, insomnia and rheumatism [11]. The medicinal effects are due to a large number of polar compounds present in the plant: phenylpropanoids glycosides (a large group of natural polyphenols, one of the best known is the verbascoside, also called acteoside), flavonoids, phenolic acids and iridoid glycosides. Verbascoside is structurally characterized by a caffeic acid moiety and a 3,4-hydroxyphenylethanol ethyl moiety (hydroxytyrosol), bound to β-(d)-glucopyranoside through ester and glycosidic links, respectively, with a rhamnose in sequence (1–3) to the glucose molecule [12].

The biological activities of *Lippia citriodora* were proven in cell culture, animal studies and a small number of human clinical trials [11]. The plant also showed a marked improvement in blood lipid profile in some animal species such as rabbit [13], hare [14], horse [15], sheep [16] and donkey [17]. However, the plant has not been fully assessed regarding its safety and efficacy in humans.

In the present study we investigated the effect of a *Lippia citriodora* leaf extract on lipid and oxidative blood profile of hypercholesterolemic adult volunteers, to identify its therapeutic benefits in human dyslipidemia.

## 2. Materials and Methods

### 2.1. Study Design and Participants

This was a preliminary, open-label, single-arm, phase I clinical study conducted on 12 hypercholesterolemic volunteers (average age 58 ± 9.02 years) whose characteristics are described in Table 1.

All subjects were evaluated by an accurate anamnesis and complete physical examination. Eligibility criteria were: age from 30 to 85 years, LDL-C > 116 mg/dL, CPK and transaminases normal values, with either an intolerance or hypersensitivity to statins. The participants also had to promise not to change eating habits and not to consume products with similar activity to *Lippia citriodora* during the study period. Exclusion criteria were: triglycerides (TG) > 500 mg/dL, type 1 and type 2 diabetes, chronic kidney disease, uncontrolled hypo- or hyperthyroidism, clinical conditions affecting the absorption of the product or adherence to the study (gastrointestinal diseases, neoplastic diseases, metabolic deficits), pregnancy or breastfeeding, a history of major cardiovascular events (acute myocardial infarction or stroke), severe peripheral atherosclerotic disease and arterial revascularization, intake of immunosuppressive agents in the previous 3 months, intake of glucocorticoids or drugs for lipid profile and body weight control and intolerance or hypersensitivity, or both, to one or more of the substances in the study. For all subjects, the 10-year risk of fatal CVD based on age, gender, smoking status, systolic blood pressure and TC (SCORE chart, low-risk regions of Europe) [5] was calculated. Each subject took a capsule containing 100 mg of *Lippia citriodora* leaf extract once a day for 16 weeks. The dosage was established based on previous animal studies [15] employing up to 1.0 mg of verbascoside per kg of metabolic body weight. Considering an average weight of 70 kg for an adult (corresponding to a metabolic weight of about 24 kg) and the percentage of phenylpropanoids (23%, consisting mainly of verbascoside) contained in the leaf extract, a 100 mg/day dose was calculated.

The study was conducted in accordance with the ethical principles stated in the Declaration of Helsinki, and the approved national and international guidelines for human research. The Institutional Review Board of the University of Molise, Campobasso, Italy, reviewed and approved this study (protocol code 006-08-2018; 2 August 2018). Written informed consent was obtained from each participant. All clinical information relating to patients has been stored and processed for statistical purposes in compliance with current privacy protection legislation.

### 2.2. Lippia citriodora Leaf Extract

*Lippia citriodora* leaf extract containing 23% phenylpropanoids (PLX23) was provided by Monteloeder (Monteloeder, Ltd., Elche, Spain). The quantitative analysis of the phenolic compounds was performed by high-performance liquid chromatography (HPLC) and reported in a manufacturer’s certificate of analysis. The plant is approved for use in humans by the Italian Ministry of Public Health, Rome, Italy.

### 2.3. Blood Collection

Blood samples were obtained from the antecubital vein, after overnight fasting, in vacutainer tubes at weeks 0, 4, 8 and 16, respectively. Timing for data collection and total treatment time were established based on previous animal studies [13,15]. The first evaluation, at 4 weeks, was carried out in order to identify any early muscular or hepatic alterations. Blood was centrifuged at 1500× *g* for 10 min. Serum was used to determine the following analytes: TC, high-density lipoprotein cholesterol (HDL-C), LDL-C, TG, glucose (GLU), CPK, aspartate aminotransferase (AST) and alanine aminotransferase (ALT), using a semiautomatic clinical chemistry analyzer (ARCO Biotecnica Instruments SPA, Rome, Italy). Oxidative state markers were measured in the plasma. Total oxidant status (TOS), which measures the blood antioxidant capacity, was determined using an assay based on the oxidation of ferrous ion to ferric ion in the presence of various oxidant species in acidic medium and ferric ion measurement by xylenol orange [18]. The results were expressed in μM H_2_O_2_ equivalent per liter. Reactive oxygen metabolites (ROM), i.e., the concentration of hydroperoxides in the plasma, were determined using a free radicals (FR) determination system (D-Roms test, Diacron International srl, Grosseto, Italy). The test is based on transition metals’ ability to catalyze in the presence of peroxides with the formation of FR, which are trapped by an alchilamine. The alchilamine reacts, forming a colored radical detectable at 505 nm [19]. The results were expressed as U Carr (1 Unit Carratelli corresponds to 0.024 mmol/L of H_2_O_2_; the higher the value measured, the greater the oxidative stress in the blood). Ferric ion reducing antioxidant power (FRAP) test measured the ferric-reducing ability of plasma. Ferric to ferrous ion reduction at low pH led to the formation of a colored ferrous-tripyridyltriazine complex. FRAP values were obtained by comparing the absorbance change at 593 nm in test reaction mixtures with those containing ferrous ions in known concentrations [20]. One FRAP unit is expressed in mmol TEAC/L (TEAC, Trolox equivalent antioxidant capacity; the higher the value obtained, the better the blood antioxidant capacity). Malondialdehyde (MDA), the ultimate product of all oxidative processes involving polyunsaturated fatty acids, was determined spectrophotometrically, according to the thiobarbituric acid (TBA) assay [21]. Vitamin E and vitamin A were extracted from plasma samples with chloroform and analyzed on an HPLC system consisting of an autosampler (HPLC Autosampler 360, Kontron Instruments, Milan, Italy) with a 20 mL loop, a high-pressure mixing pump and a 5 µm, 250 × 4.60 mm C18 column (Phenomenex, Torrance, CA, USA). The mobile phase was 100% methanol at a flow rate of 1.0 mL/min. A fluorimeter detector (SFM) and computer with Kroma System 2000 software were used. The concentrations of vitamins A and E were determined by using an internal standard and the elution time of pure standards [22].

### 2.4. Statistical Analysis

Data were analyzed using SPSS (v. 17.0) statistical software package (SPSS Inc., Chicago, IL, USA). Variables were examined for outliers and extreme values using box and normal quantile-quantile plots, and Shapiro—Wilk’s and Kolmogorov—Smirnov’s tests. No variables needed to be transformed. Repeated measures analysis of variance (ANOVA) and post hoc pairwise comparison tests, namely least significant difference (LSD) and Bonferroni’s correction (BC), were used. The assumptions of sphericity were assessed by means of Mauchly’s test. The Greenhouse—Geisser or the Huynh—Feldt correction was applied if the assumption of sphericity was violated. Statistical significance was set at *p* value < 0.05.

## 3. Results

Using repeated measures ANOVA, the lipid blood profile showed a weak significant reduction in TC (*p* = 0.035) and a strong significant increase in HDL-C (*p* = 0.007) levels during treatment with *Lippia citriodora* leaf extract, as reported in Table 2.

**Figure 1:**
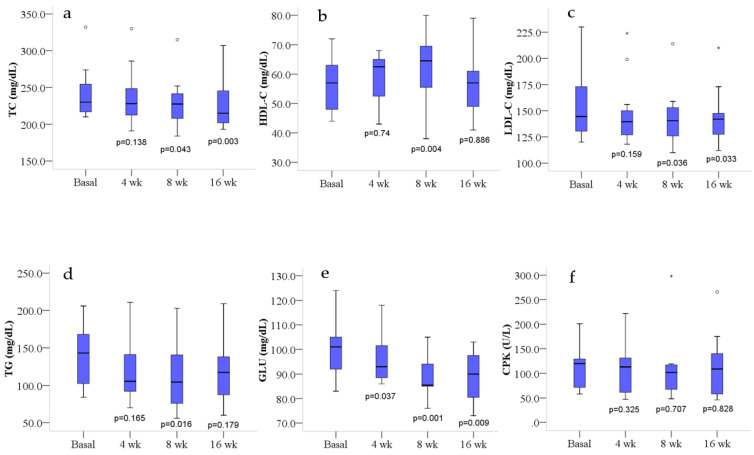
Lipids, glucose and CPK blood profile at basal level and after 4, 8 and 16 weeks of treatment. Box plots show median (horizontal line in the box), 25th and 75th percentiles (edges of box), maximum and minimum values (whiskers) and outliers (°, *) of: (**a**) total cholesterol (TC), (**b**) high-density lipoprotein cholesterol (HDL-C), (**c**) low-density lipoprotein cholesterol (LDL-C), (**d**) triglycerides (TG), (**e**) glucose (GLU) and (**f**) creatine phosphokinase (CPK) concentrations. *p* values measure the significance of differences compared with basal values (least significant difference); wk, weeks.

Post hoc LSD pairwise comparisons showed a weak reduction in TC at 8 weeks (*p* = 0.043), not significant after BC, and a strong reduction (*p* = 0.016, after BC) at 16 weeks of treatment (Figure 1a). HDL-C significantly increased (*p* = 0.026, after BC) only after 8 weeks of treatment (Figure 1b). LDL-C showed a reduction at the limit of significance (*p* = 0.072) during the treatment (Table 2). Post hoc LSD pairwise comparison (Figure 1c) showed a weak reduction at 8 (*p* = 0.036) and 16 weeks (*p* = 0.033), not significant after BC. TG levels showed a not significant downward trend after treatment (Table 2). However, post hoc LSD pairwise comparison (Figure 1d) showed a fairly good reduction at 8 weeks (*p* = 0.016), not significant after BC (*p* = 0.098). GLU levels showed a significant reduction (*p* = 0.002) during the treatment (Table 2), particularly at 8 and 16 weeks (Figure 1e). However, the reduction at 16 weeks was at the limit of significance (*p* = 0.056) after BC.

**Figure 2:**
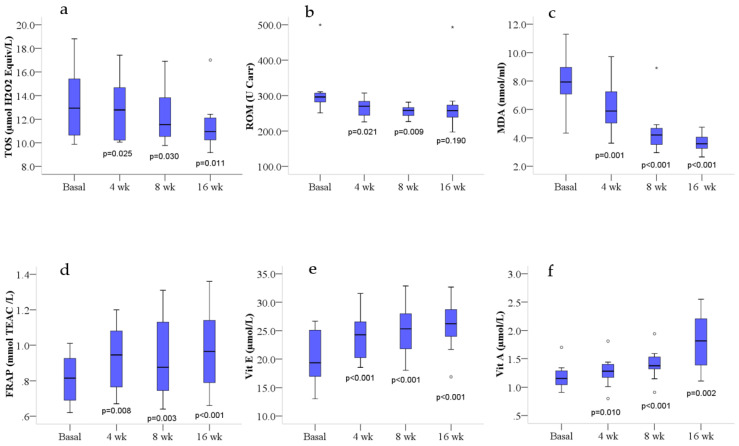
Blood oxidative profile at basal level and after 4, 8 and 16 weeks of treatment. Box plots show median (horizontal line in the box), 25th and 75th percentiles (edges of box), maximum and minimum values (whiskers) and outliers (°, *) of: (**a**) total oxidant status (TOS), (**b**) reactive oxygen metabolites (ROM), (**c**) malondialdehyde (MDA), (**d**) ferric reducing ability of plasma (FRAP), (**e**) vitamin E (Vit E) and (**f**) vitamin A (Vit A) concentrations. *p* values measure the significance of differences compared with basal values (least significant difference); wk, weeks.

The results related to the oxidative state markers differences during the treatment with *Lippia citriodora* leaf extract showed a global improvement of the oxidative blood profile. In fact, TOS levels were significantly reduced (*p* = 0.012, after correction for lack of sphericity), after treatment. Post hoc LSD pairwise comparisons showed a reduction at 4, 8 and 16 weeks (Figure 2a); however, only the reduction at 16 weeks was at the limit of significance (*p* = 0.068) after BC. ROM levels were weakly reduced (*p* = 0.069, after correction for lack of sphericity), after treatment. Post hoc LSD pairwise comparisons showed a weak reduction at 4 weeks (*p* = 0.021), not significant after BC, and a stronger reduction (*p* = 0.009) at 8 weeks of treatment (Figure 2b), which was at the limit of significance (*p* = 0.055) after BC. MDA levels were significantly decreased (Table 2) at 4, 8 and 16 weeks of treatment (Figure 2c), even after BC. Conversely, FRAP, vitamin E and vitamin A levels were significantly increased (Table 2) at 4, 8 and 16 weeks of treatment (Figure 2d–f), even after BC, with the exception of FRAP and vitamin A levels at 4 weeks which were at the limit of significance (*p* = 0.050 and *p* = 0.061, respectively). LSD pairwise comparison did not show significant differences between women and men for any of the measured parameters.

To highlight possible adverse events concerning the intake of the extract, some analytes were also measured to check for potential organ damage (AST, ALT), as well as the serum levels of CPK (whose values increase in patients taking statins) to evaluate problems affecting skeletal muscle tissue. No significant variations were observed regarding the CPK and transaminase (AST and ALT) levels (Table 2 and Figure 1f), and no side or adverse effects were reported by participants.

## 4. Discussion

In the present study, we analyzed the lipid and oxidative profile changes induced by treatment with *Lippia citriodora* leaf extract (100 mg, once a day, for 16 weeks) in 12 hypercholesterolemic volunteers (7 males and 5 females). Results showed an improvement in blood lipid profile, compared with baselines, with a significant decrease in TC, particularly at 16 weeks, and a significant increase in HDL-C at 8 weeks. Additionally, TG and LDL-C showed a downward trend at 8 weeks and at both 8 and 16 weeks, respectively. These results are broadly in line with previous studies reporting significant changes on the lipid profile after dietary supplementation with a *Lippia citriodora* extract. Rabbits [13], hares [14], horses [15] and sheep [16] treated with low and high doses of verbascoside showed a decrease in TC, LDL-C, (not measured in the hare study), TG and an elevation in HDL-C compared with control groups, who received a feed without verbascoside. Higher doses of dietary supplementation with verbascoside generally produced better effects than lower doses did, but the differences were not statistically significant. Yang et al. [23] evaluated the effects of *Ligustrum robustum* Blume (LR), a plant used in Chinese folk medicine for the treatment of obesity and hyperlipidemia, in mice fed with a high-fat diet. LR leaf extracts, mainly containing phenylpropanoid glycosides with verbascoside as a major active component, produced a significant reduction in TC and a downward trend in TG levels, without a significant dose—effect relation. Kassi et al. [24] carried out a randomized placebo-controlled trial in normocholesterolemic healthy volunteers. Following the administration of an aqueous extract of *Sideritis euboea* (containing verbascoside, flavonoid diglycosides and phenolic acid), at 0.3 g/day for a month, the intervention group showed a significant reduction in TC, compared with the control group.

The hypocholesterolemic effect of the *Lippia citriodora* extract can be explained by some of the molecular mechanisms related to verbascoside, namely a downregulation of the mRNA-encoding enzymes involved in cholesterol biosynthesis, such as HMG-CoA reductase and mevalonate kinase, and an upregulation of the mRNA encoding molecules involved in lipid transport and metabolism, such as VLDL receptor, lipoprotein lipase (LPL), lipin 1, peroxisome proliferator activated receptor-alpha (PPARα), acetyl CoA acyl transferases (Acaa1a and Acaa1b) and carnitine palmitoyl transferase 1A (Cpt1a) [25]. PPARα is known to be able to modulate the production of the apoproteins ApoA-1 and ApoA-2 (the main constituents of HDL-C) and this could be directly related to HDL-C concentration [26]. Furthermore, using HepG2 cells treated with a high concentration of oleic acid as a lipid accumulation model, it has been shown that verbascoside is also able to downregulate the expression of 7-dehydrocholesterol reductase (DHCR7), lanosterol synthase (LSS) and farnesyl-diphosphate farnesyltransferase 1 (FDFT1), which are involved in cholesterol biosynthesis, and to increase the expression of HDL scavenger receptors class B member 1 and 2 (SCARB1 and SCARB2) [27]. SCARB1 is the primary receptor involved in the transfer of circulating cholesterol from HDL to the liver, termed as the reverse cholesterol transport pathway, ending with the excretion of cholesterol via bile and feces [28].

According to these findings, we can assume that the TC and LDL-C decrease seen in our study is probably due to a reduction in cholesterol biosynthesis and to an increase in lipid β-oxidation, whereas the HDL-C elevation at 8 weeks is likely related to PPARα upregulation. The return of HDL-C levels to baseline values at 16 weeks may be caused by overexpression of SCARB1, which causes an increased hepatic metabolism and clearance of HDL-C [28]. However, the reduction in circulating HDL-C seems to have a beneficial effect against atherosclerosis. Indeed, whole-body SCARB1 knockout (KO) (Sr-bl^−/−^) mice showed an increase in HDL-C with a significant rise in aortic lesions, proving that SCARB1 deficiency is proatherogenic. Conversely, overexpression of SCARB1 in mouse models reduces atherosclerosis [28].

The increased PPARα and LPL expression may also explain the downward trend in TG in the present study [26].

Verbascoside also prevents the formation of oxidized LDL-C (oxLDL), lowers the expression of genes related to oxidative stress and oxLDL-mediated inflammatory response and protects endothelial cells from oxLDL-induced cytotoxicity [29]. The accumulation of oxLDL in the arterial intima contributes significantly to the recruitment of monocytes and the formation of “foam cells” (early atherosclerotic lesions composed of cholesterol-engorged macrophages) [4]. Considering the crucial role of oxLDL in the pathogenesis of atherosclerotic plaques, we can presume that circulant LDL-C in subjects treated with *Lippia citriodora* is less atherogenic as it is less oxidized.

Treatment with *Lippia citriodora* leaf extract in the present study also improved the oxidative status with a significant decrease in TOS and MDA, a downward trend in ROM and a significant increase in FRAP, vitamin E and vitamin A. Carrera-Quintanar et al. [30] underlined the benefits of *Lippia citriodora* extract on the oxidative stress induced by aerobic training in university students. Indeed, verbascoside was able to increase the activity of the main antioxidant enzymes like catalase, glutathione peroxidase and glutathione reductase [30], probably acting at the post-transcriptional level or through a Nrf2-related mechanism [29]. A marked improvement in blood oxidative status has also been detected in some animal species, such as rabbit [13], hare [14], horse [15], sheep [16] and donkey [17], resulting in a decrease in plasma ROM and thiobarbituric acid-reactive substances values, and an increase in the concentrations of plasma vitamin E and vitamin A. Generally, high doses of verbascoside produced better effects than low doses, but the differences were not statistically significant.

Similarly, a study using animal models showed a decrease in oxidative stress-correlated enzyme activity in the plasma, brain and hippocampus, as well as a decrease in cerebral MDA, after verbascoside administration [31]. The elevation of vitamin E and vitamin A, both concurring to the improvement of blood antioxidant power [32], is likely due to verbascoside influence on the stability of low molecular weight molecules such as these vitamins [15]. Vitamin A (through retinol and β-carotene) and vitamin E (through α-Tocopherol) protect cell membranes from lipid peroxidation, acting as chain-breaking antioxidants. They also act as scavengers of hydroxyl radicals, superoxide anions and peroxynitrite [32].

Flavonoids and phenolic acids present in *Lippia citriodora* may also be responsible for the antioxidant phenotype [33]. The enhanced blood antioxidant capacity is beneficial for endothelial function, since oxidant stimuli cause an imbalance in vasoconstrictor, prothrombotic, proliferative and inflammatory pathways and this phenotype is characteristic of chronic inflammatory diseases such as atherosclerosis [34]. Some studies show that verbascoside also has a vasodilator action, inhibiting the contractions induced by noradrenaline [35], and an anti-inflammatory effect since it is able to inhibit downstream proinflammatory cytokines and growth factors [29,36].

Another beneficial effect of *Lippia citriodora* treatment in the present study was the significant improvement of the glycemic profile. Indeed, verbascoside can suppress postprandial glucose peak and normalize glucose tolerance, likely due to inhibition of sodium-dependent glucose co-transporter 1 (SGLUT-1). In addition to verbascoside, iridoids present in the extract also have an antidiabetic activity [35].

Overall, the results of this study suggest that *Lippia citriodora*, through its compounds such as verbascoside, flavonoids, phenolic acids and iridoids, has pleiotropic effects similar to statins; it acts not only on lipid profile, but also on lipoprotein oxidation, endothelial function and inflammatory status. Its notable polypharmacological effects could represent a valuable nutraceutical approach for the management of hypercholesterolemia and the prevention of CVD. Unlike statins, however, the safety and tolerability profile of *Lippia citriodora* treatment seems more favorable, since transaminases and CPK were not altered and no subjects reported side or adverse effects. These findings are in line with previous studies showing that supplementation with *Lippia citriodora* extract decreases the signs of muscular damage in chronic running exercise, without blocking the cellular adaptation to exercise [37].

We are aware that the present study has several limitations: the number of participants is small, the study is open-label, a control group is missing and follow-up information is lacking. This is motivated by the fact that our intention was to perform a pilot, preliminary study aimed at verifying whether the beneficial effects of *Lippia citriodora* on the lipid and oxidative blood profile demonstrated in previous animal studies were also present in humans. A randomized, placebo-controlled, multiple dose, double-blind clinical trial, eventually including sex- and age-matched participants without hypercholesterolemia, is certainly needed to evaluate the safety and efficacy of this plant extract.

## 5. Conclusions

In conclusion, this preliminary study demonstrated that dietary supplementation with *Lippia citriodora* extract can improve the lipid profile, particularly by reducing TC and increasing HDL-C, and enhance the blood antioxidant power, particularly by reducing TOS and MDA and increasing FRAP and vitamins A and E. These results suggest that *Lippia citriodora* could be a valuable natural compound for the management of dyslipidemia and the prevention of CVD. Clinical trials of phase II and III are necessary to confirm these findings.

## Figures and Tables

**Table 1:** Baseline characteristics of the analyzed subjects.

ID	SEX	AGE (Years)	SMOKE	BMI (kg/m^2^)	GLU (mg/dL)	BP (mmHg)	TC (mg/dL)	LDL-C (mg/dL)	SCORE
1	M	60	No	24.8	104	135/85	212	140.00	3%
2	M	68	Yes	25.4	102	150/90	210	120.00	13%
3	M	65	Yes	23.3	124	120/80	236	131.00	7%
4	M	63	No	22.9	101	120/70	220	129.00	3%
5	F	44	No	34.2	83	120/70	261	168.00	0%
6	M	57	Yes	44.9	91	140/90	229	168.00	5%
7	F	59	Yes	26.9	106	130/80	274	180.00	3%
8	M	67	No	24.4	110	140/80	214	149.00	6%
9	M	53	Yes	23.6	93	120/80	224	134.00	2%
10	F	56	No	25.8	97	120/80	231	130.00	1%
11	F	39	No	33.3	101	125/70	332	230.00	0%
12	F	65	Yes	20.1	90	150/110	248	178.00	7%

M, male; F, female; BMI, body mass index; GLU, glucose; BP, blood pressure; TC, total cholesterol; LDL-C, low-density lipoprotein cholesterol; SCORE, Systematic Coronary Risk Evaluation.

**Table 2:** Blood parameters of 12 volunteers at baseline and after 4, 8 and 16 weeks of *Lippia citriodora* extract treatment.

	BASELINE	4 WEEKS	8 WEEKS	16 WEEKS	ANOVAF (df = 3, 33)	*p* Value
TC	240.9 ± 10.1	236.2 ± 11.4	229.2 ± 9.9 *	227.0 ± 10.2 *	3.22	0.035
HDL-C	56.6 ± 2.7	59.1 ± 2.3	62.7 ± 3.3 *	56.8 ± 3.2	4.78	0.007
LDL-C	154.7 ± 9.1	147.7 ± 9.2	143.7 ± 7.7 *	144.1 ± 7.6 *	2.56	0.072
TG	139.8 ± 11.3	121.4 ± 12.6	111.8 ± 12.3 *	119.8 ± 13.1	2.10	0.119
GLU	100.2 ± 3.1	96.3 ± 2.8 *	88.7 ± 2.3 *	89.4 ± 2.9 *	10.11	0.002
CPK	111.7 ± 12.2	107.2 ± 14.9	107.7 ± 18.9	113.7 ± 18.4	0.32	0.321
AST	25.7 ± 2.9	21.8 ± 1.6	23.2 ± 2.8	21.0 ± 1.8	0.96	0.423
ALT	30.7 ± 5.3	24.8 ± 4.2	26.9 ± 3.6	22.3 ± 1.6	1.78	0.169
TOS	13.4 ± 0.9	12.9 ± 0.7 *	12.3 ± 0.6 *	11.5 ± 0.6 *	7.42	0.012
ROM	306.5 ± 18.3	267.8 ± 7.6 *	255.3 ± 4.9 *	271.4 ± 21.3	2.99	0.069
MDA	8.1 ± 0.5	6.1 ± 0.5 *	4.4 ± 0.4 *	3.7 ± 0.2 *	35.23	<0.001
FRAP	0.8 ± 0.04	0.9 ± 0.05 *	0.9 ± 0.06 *	0.9 ± 0.06 *	8.13	0.002
Vit E	20.3 ± 1.4	23.9 ± 1.2 *	25.2 ± 1.3 *	26.2 ± 1.3 *	27.08	<0.001
Vit A	1.2 ± 0.06	1.3 ± 0.07 *	1.4 ± 0.07 *	1.8 ± 0.14 *	13.88	0.002

TC, total cholesterol (mg/dL); HDL-C, high-density lipoprotein cholesterol (mg/dL); LDL-C, low-density lipoprotein cholesterol (mg/dL); TG, triglycerides (mg/dL); GLU, glucose (mg/dL); CPK, creatine phosphokinase (U/L); AST, aspartate transaminase (U/L); ALT, alanine transaminase (U/L); TOS, total oxidant status (µmol H_2_0_2_ Eq/L); ROM, reactive oxygen metabolites (U Carr); MDA, malondialdehyde (nmol/mL); FRAP, ferric reducing ability of plasma (mmol TEAC/L); Vit E, vitamin E (µmol/L); Vit A, vitamin A (µmol/L). Values are mean ± standard deviation; * significantly different compared with baseline (least significant difference).

## Data Availability

All data are presented in the paper.
